# Interleukin-33 amplifies IgE synthesis and triggers mast cell degranulation via interleukin-4 in naïve mice^1^

**DOI:** 10.1111/j.1398-9995.2012.02859.x

**Published:** 2012-06-15

**Authors:** M Komai-Koma, F Brombacher, P N Pushparaj, B Arendse, C McSharry, J Alexander, R Chaudhuri, N C Thomson, A N J McKenzie, I McInnes, F Y Liew, D Xu

**Affiliations:** 1 Institute of Infection, Immunity and Inflammation, University of GlasgowGlasgow, UK; 2 Institute of Infectious Disease and Molecular Medicine, University of Cape Town and International Center for Genetic Engineering and BiotechnologyCape Town, South Africa; 3 Center of Excellence in Genomic Medicine Research, King Abdulaziz UniversityJeddah, Saudi Arabia; 4 Strathclyde Institute of Pharmacy and Biomedical Sciences, University of StrathclydeGlasgow, UK; 5 Medical Research Council Laboratory of Molecular BiologyCambridge, UK

**Keywords:** allergen independent, IgE, IL-33, IL-4 dependent, mast cell degranulation

## Abstract

**Background:**

The regulation and function of IgE in healthy individuals and in antigen-naïve animals is not well understood. IL-33 administration increases serum IgE in mice with unknown mechanism. We tested the hypothesis that IL-33 provides an antigen-independent stimulus for IgE production and mast cell degranulation.

**Methods:**

IL-33 was administered to naïve wild-type (WT), nude and ST2^−/−^, IL-4^−/−^, IL4Rα^−/−^ and T-or B-cell-specific IL-4Rα^−/−^ mice. IgEand cytokines were quantified by ELISA. T- and B-lymphocyte numbers and CD40L expression were determined by flow cytometry. Anaphylaxis was measured by temperature, mast cell degranulation and histamine release.

**Results:**

IL-33 enhanced IgE production in naïve WT, T-IL-4Rα^−/−^ but not in ST2^−/−^, IL-4^−/−^, IL-4Rα^−/−^ or B-cell-specific IL-4Rα^−/−^ mice, demonstrating IL-33 specificity and IL-4 dependency. Moreover, IL-4 was required for IL-33-induced B-cell proliferation and T-cell CD40L expression, which promotes IgE production. IL-33-induced IL-4 production was mainly from innate cells including mast cells and eosinophils. IL-33 increased mast cell surface IgE and triggered degranulation and systemic anaphylaxis in allergen-naïve WT but not in IL-4Rα^−/−^ mice.

**Conclusion:**

IL-33 amplifies IgE synthesis and triggers anaphylaxis in naïve mice via IL-4, independent of allergen. IL-33 may play an important role in nonatopic allergy and idiopathic anaphylaxis.

IgE antibody is induced by allergen and requires immunoglobulin heavy-chain class switching and affinity maturation in B cells in a T-cell-dependent manner 1, 2. IgE is also produced naturally 1, 3 but less is known about the selective amplification and function of natural IgE in healthy subjects and antigen-naïve animals 3. Natural IgE is secreted by B2 cells and contributes to the baseline concentrations of nonallergen-specific IgE in the serum and tissues of healthy individuals 3, 4. In contrast to allergen-induced IgE, cognate T-helper cell via MHC class II and somatic hypermutation are not required for the generation of natural IgE 3. Instead, bystander stimulation from T-cell CD40L and low levels of IL-4 are necessary 3, 4. The functions of natural IgE are poorly understood; they are polyreactive and have a critical role in immune priming 3–7. IgE can bind the high-affinity IgE receptor (FcεRI) and prolong mast cell survival, release a spectrum of cytokines and have important roles in defence against infection and initiation of adaptive immunity 5–8.

IL-33 is a new IL-1 family member and signals via a receptor complex consisting of ST2 (IL-1LR1) and IL-1RAcP 9, 10. IL-33 is expressed by innate cells in humans and mice, primarily epithelium and endothelium, and can be released when cells sense inflammatory signals or undergo necrosis 9–11. ST2 is expressed by most innate cells but only by selected adaptive immune cells 9, 12, 13. Thus, IL-33 can directly activate eosinophils, basophils, macrophages and NK cells via ST2 9, 14–16. In contrast, ST2 is selectively expressed on IL-5^+^Th, Th2 but not Th1 or Th17 cells 17–19. While enhancing mature Th2-cell function, IL-33 cannot polarize Th2 cells 17. Interestingly, IL-33 can induce IgE production in naïve mice 9. However, the effect and mechanism by which IL-33 induces IgE production in naïve mice is largely unknown.

We aim to identify the mechanism and role of IL-33 in the induction and regulation of IgE production in allergen-naïve mice. We report that IL-33 amplifies IgE synthesis via IL-4 from innate immune cells that is sufficient to trigger mast cell degranulation and anaphylaxis in the absence of allergen.

## Materials and methods

### Mice

BALB/c, BALB/c nude and C57BL/6 mice were purchased from Harlan Olac. ST2^−/−^ mice 20, IL-4^−/−^, IL-4 receptor α chain knockout (IL-4Rα^−/−^) 21 and mice selectively deficient in IL-4Rα on B cells (F. Brombacher, unpublished) or T cells (*Lck*^*cre*^*Il4ra*^*−/lox*^) 22 were on a BALB/c background. Mice were housed in specific-pathogen-free conditions at Glasgow University, UK, and Cape Town University, South Africa, in accordance with animal experimentation guidelines.

### IL-33 treatment *in vivo*

Recombinant IL-33 was prepared as described 23. Mice were injected i.p. with IL-33 (2 μg/mouse) or PBS daily for 1–7 days unless stated otherwise. Cells were collected by peritoneal washout with 2 ml of cold PBS.

Anaphylaxis (AS): Mice were injected i.p. with IL-33 (2 μg) or PBS daily for 3–4 days. Changes in clinical signs and temperature were monitored every 10 min after each injection, using a rectal probe (Natsume Seisakusyo Co., Tokyo, Japan). Plasma histamine was measured by ELISA (IBL, Hamburg, Germany).

### Flow cytometry

Cells (3 × 10^5^ cells/tube) were stained with fluorochrome-conjugated antibodies anti-CD3, anti-CD4, anti-CD19, anti-FcεRI, anti-CD40L, anti-CD25, anti-CD117 and Siglec-F and isotype controls (BD Bioscience, Oxford, UK). Intracellular cytokine levels in peritoneal cells were determined by staining with anti-IL-4-PE after activation (PMA/ionomycin) and permeabilization. Cells were analysed by FACSCalibur using Cell Quest software (BD Bioscience).

### Cytokine and IgE measurements

Serum concentrations of cytokines were determined by Luminex (Luminex, Biosource; Invitrogen, Life Technologies Ltd, Paisley, UK) and IgE by ELISA (Bethyl Laboratories, Inc, Montgomery, TX, USA).

### Histological analysis of mast cells

Skin samples were stained with toluidine blue to identify and count mast cells by microscopy. Degranulated mast cells were identified as those with >10% extrusion of granules 20.

### Statistical analysis

Student's *t*-test was applied to *in vitro* studies. Analysis between individuals in groups *in vivo* was carried out by anova followed by Student's *t*-test.

## Results

### IL-33-induced IgE but not type-II cytokines is IL-4 dependent

**Figure 1:**
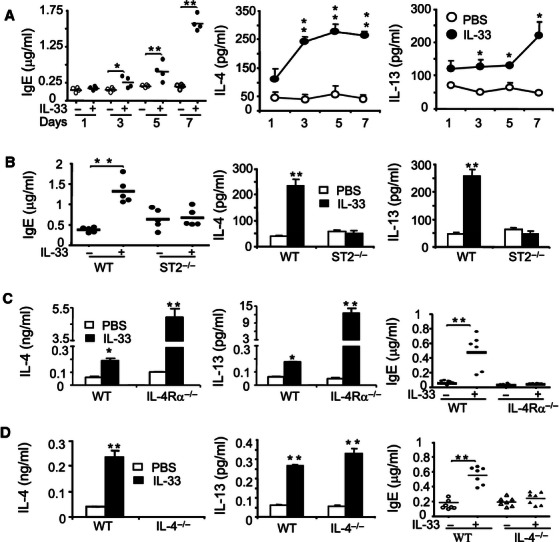
IL-33 induces IgE but not type-II cytokine production in an IL-4-dependent manner. Groups of wild-type (A), ST2^−/−^ (B), IL-4Rα^−/−^ (C) and IL-4^−/−^ (D) mice were injected i.p. with IL-33 (2 μg) or PBS daily for 7 days. Total serum IgE, IL-4 and IL-13 concentrations were measured by ELISA. Data are representative of three experiments, *n* = 5–7 mice per group, *P < 0.05, **P < 0.01 compared to PBS controls.

Mice had seven daily injections of IL-33 or PBS. Low basal concentrations of serum IgE, IL-4 and IL-13 were maintained in antigen-naïve or PBS-treated mice, but were significantly elevated in wild-type (WT), but not ST2^−/−^ mice, within 3 days of IL-33 administration (Fig. 1A,B) IL-4 production preceded the increase in IgE. As reported 9, IL-33 did not induce IFN-γ in naïve WT mice (data not shown). These results suggest that IL-33 is a powerful and rapid inducer of IgE in naïve mice that might be mediated by type-II cytokines.

IL-4 in mice and IL-4 and IL-13 in humans are key cytokines for the initiation of type-II responses and IgE isotype class switching 4, 24. We therefore determined the role of IL-4 and IL-13 in IL-33-induced IgE synthesis. WT and IL-4Rα^−/−^ mice were injected with IL-33 resulting in higher serum concentrations of IL-4 and IL-13 in IL-4Rα^−/−^ mice compared with PBS-treated IL-4Rα^−/−^ mice or with IL-33-treated WT mice (Fig. 1C), probably due to reduced sequestration of these cytokines in the absence of IL-4Rα 25. More importantly, IL-33 failed to enhance IgE in IL-4Rα^−/−^ mice (Fig. 1C). Together, these data indicate that IL-33-mediated induction of IgE is dependent on IL-4Rα responsiveness and that IL-4/IL-13 induction is not.

Because IL-4Rα deficiency abolishes both IL-4 and IL-13 functions 21, 22, we assessed the requirement of IL-4 for IgE production using IL-4^−/−^ mice. WT and IL-4^−/−^ mice were injected with IL-33. IL-33 induced similar levels of IL-13 production in the WT and IL-4^−/−^ mice (Fig. 1D), but IL-33 failed to enhance IgE in IL-4^−/−^ mice (Fig. 1D). Thus, IL-4 is necessary and sufficient for the induction of IgE by IL-33 in naïve mice.

### IL-33 induces type-II cytokines mainly via innate cells in naïve mice

**Figure 2:**
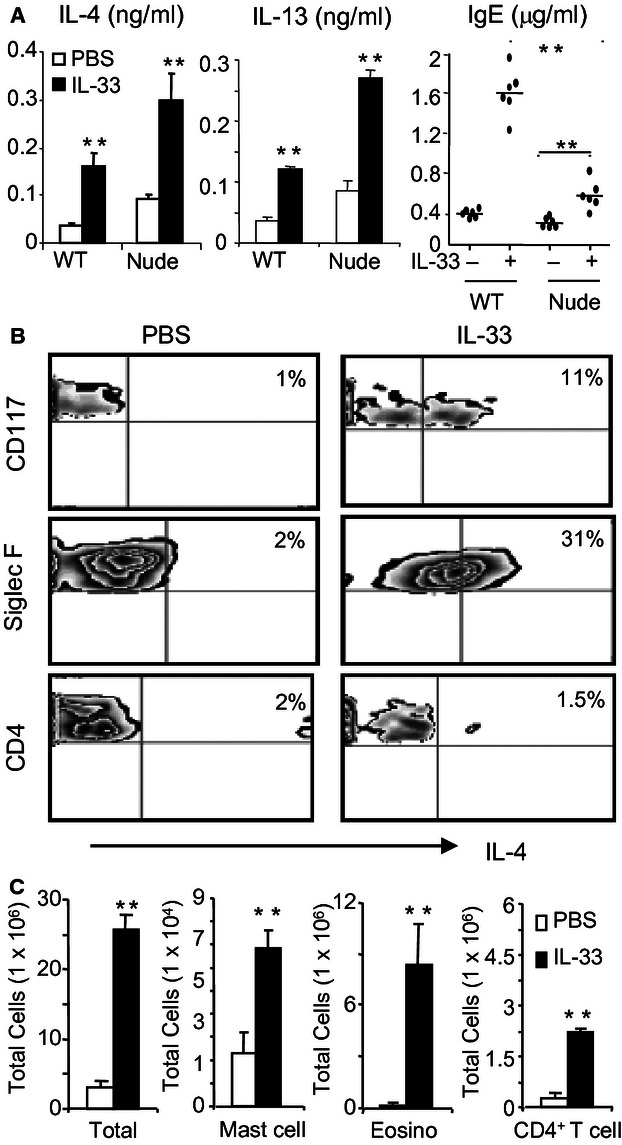
IL-33 induces type-II cytokines mainly via innate cells in naïve mice. Mice were treated with IL-33 for 7 days. Serum IL-4, IL-13 and IgE concentrations in (A) nude and wild-type (WT) mice were measured by ELISA. (B) The frequency of IL-4^+^ cells and (C) total numbers of different cell populations in the peritoneum of WT mice treated with IL-33 or PBS were determined by intracellular staining using FACScan and quantified. Data are the representative of two experiments, *n* = 5 mice per group, *P < 0.05, **P < 0.01 compared to the PBS control or as indicated.

We next investigated whether the increased IL-4 and IL-13 levels in IL-33-treated mice were produced by T cells or innate cells *in vivo* by comparing WT and athymic nude mice (lacking T cells) injected with IL-33 or PBS as described above. Nude mice produced significantly higher levels of serum IL-4 and IL-13 than IL-33-treated WT mice, but markedly lower IgE (Fig. 2A). This suggests two points. First, T cells have minimal contribution to IL-33-induced type-II cytokine production *in vivo*. Second, while IL-33 could induce significant amounts of IgE in nude mice compared with PBS control, the levels of IgE were markedly lower than those of IL-33 in WT mice, suggesting that T cells have a modest but significant role in IL-33-mediated IgE induction, independent of enhanced type-II cytokine synthesis.

We next determined which were the IL-4-producing cells *in vivo* from IL-33- or PBS-treated WT mice by flow cytometry. IL-33 enhanced the IL-4 expression in CD117 (c-Kit)^+^ mast cells (11% compared with 1% in PBS-treated controls) and in Siglec-F^+^ eosinophils (31% compared with 2% in PBS controls) (Fig. 2B). However, IL-33 failed to increase the frequency of IL-4^+^ CD4^+^ T cells (Fig. 2B). IL-33 also increased the numbers of total cells, mast cells, eosinophils and to a lesser extent CD4^+^ T cells in the peritoneal cavity (Fig. 2C). Thus, IL-33 mainly induces type-II cytokines via innate immune cells in naïve mice.

### B cells expressing the IL-4Rα are required for IL-33-induced B-cell expansion and IgE production *in vivo*

**Figure 3:**
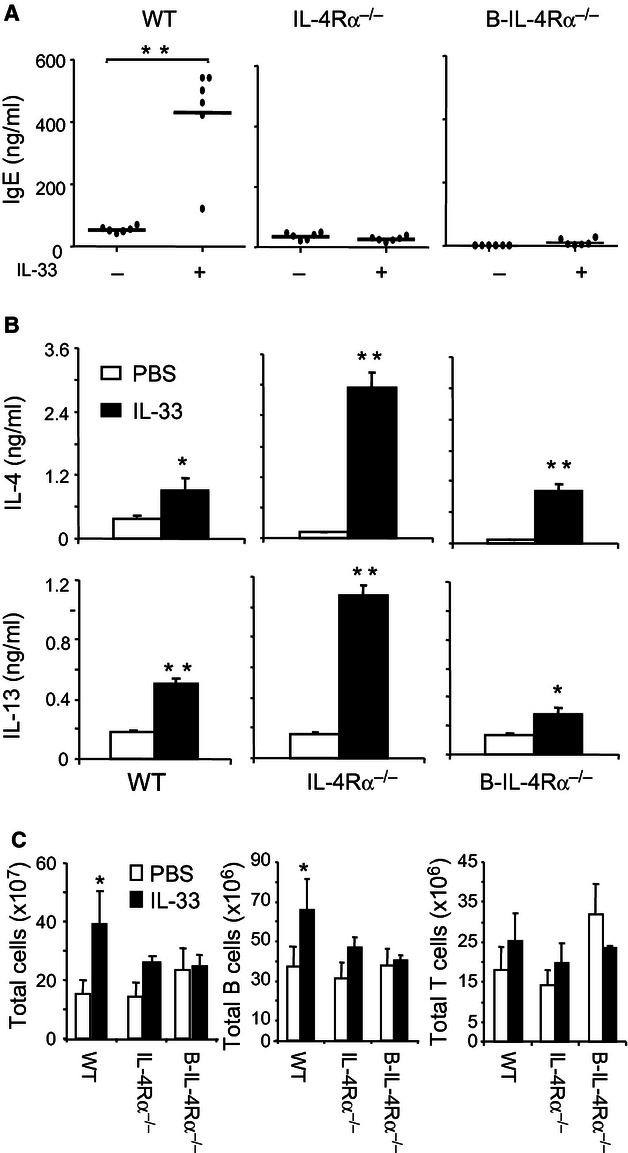
B cells expressing IL-4Rα are required for IL-33-induced B-cell expansion and IgE production *in vivo*. Wild-type, IL-4Rα and B-IL-4Rα mice were injected with IL-33. The levels of serum IgE (A) and IL-4/IL-13 (B) were measured by ELISA. (C) Total cell number, T cells and B cells in the spleen were determined by differential cell count. Data are the representative of two experiments, *n* = 5 mice per group, *P < 0.05, **P < 0.01 compared to PBS group.

We have previously shown that conventional CD19^+^ B cells do not express ST2 26, suggesting that IL-33 may only induce IgE synthesis indirectly, likely via IL-4 as we have demonstrated earlier. We therefore assessed the role of IL-4Rα signalling on B cells in IL-33-induced IgE synthesis by comparing naïve B-cell-specific IL-4Rα^−/−^ (selectively deleted of IL-4Rα on B cells), IL-4Rα^−/−^ (total IL-4Rα deletion) and WT mice treated with IL-33 or PBS. IL-33 induced IgE synthesis in WT but not in IL-4Rα^−/−^ or B-IL-4Rα^−/−^ mice (Fig. 3A). Serum IL-4 and IL-13 concentrations in B-IL-4Rα^−/−^ and WT mice were similar but were significantly lower than those of the IL-4Rα^−/−^ mice (Fig. 3B), suggesting that cells other than B cells may consume some of these cytokines. IL-33 increased the total cell number and the number of CD19^+^ B cells (but not CD4^+^ T cells) in the spleen of WT mice but not in IL-4Rα^−/−^ or B-IL-4Rα^−/−^ mice (Fig. 3C). Because IL-13 does not directly induce IgE in mouse B cells 24, together these results suggest that IL-33 indirectly elicits natural IgE production in naïve mice by inducing IL-4, which then directly stimulates B-cell expansion and IgE isotype class switching.

### T cells expressing IL-4Rα contribute to IL-33-induced IgE synthesis

**Figure 4:**
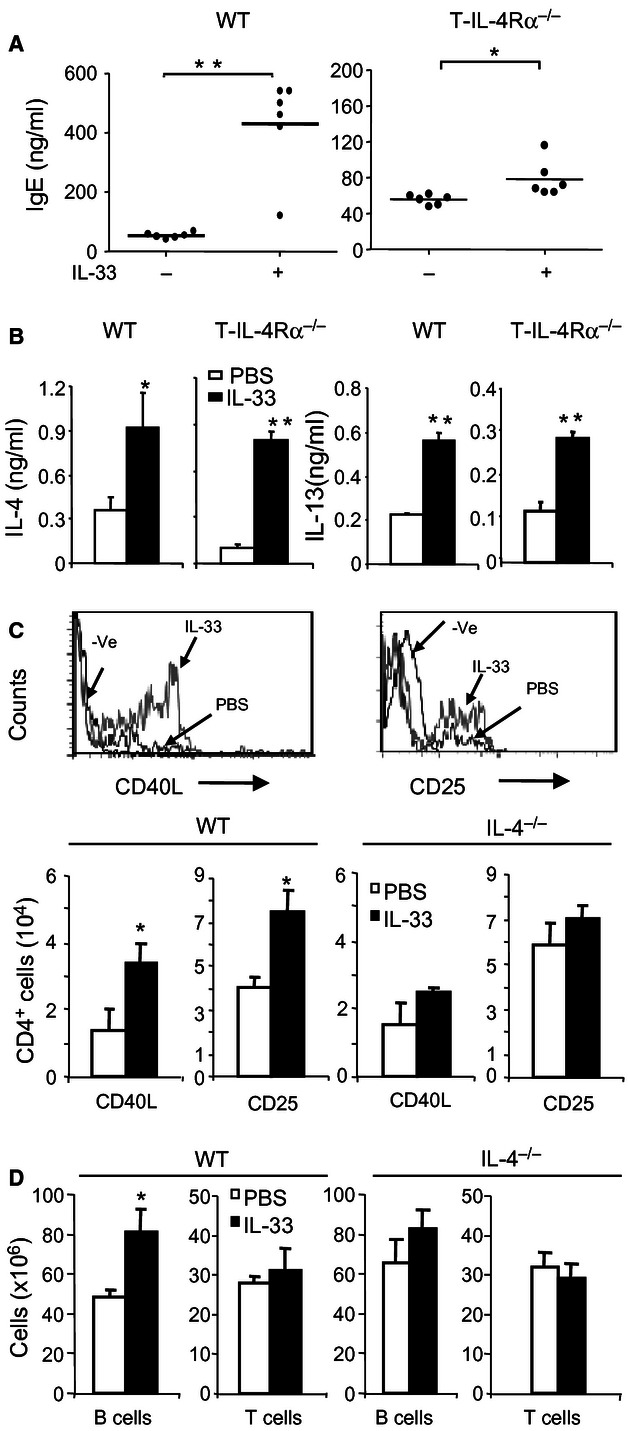
IL-4Rα T cells contribute to IL-33-induced IgE synthesis. Wild-type (WT), IL-4^−/−^ or T-IL-4Rα^−/−^ mice were treated with IL-33. (A) Serum IgE and (B) IL-4 and IL-13 concentrations were measured by ELISA. (C) The levels of CD40L and CD25 on CD4^+^ T cells and the number of CD4^+^ CD40L^+^, CD4^+^ CD25^+^ T cells in the spleen of WT and IL-4^−/−^ mice were determined by FACScan and differential counting. (D) Total CD4^+^ T and CD19^+^ B cells in the spleen of WT and IL-4^−/−^ mice were determined by differential cell count. Data are from two experiments, *n* = 6 mice per group, *P < 0.05, **P < 0.01 compared to PBS control.

Our aforementioned results (Fig. 2A,B) suggest that T cells also contribute to IL-33-enhanced IgE production in naïve mice other than by providing type-II cytokines. To assess the potential role of IL-4R responses by T cells, we injected IL-33 or PBS into WT or T-IL4Rα^−/−^ (*Lck*^*cre*^*Il4r*α^*−/lox*^ mice, selectively deleted of IL-4Rα on T cells) mice. IL-33 modestly but significantly increased serum IgE levels in T-IL-4Rα^−/−^ mice (Fig. 4A). Consistent with the result from nude mice (Fig. 2A), IL-33 elicited abundantly increased level of IL-4 and IL-13 in T-IL4Rα^−/−^ compared with WT mice (Fig. 4B), suggesting that these cytokines are unlikely to play an important role in T-cell function in IL-33-induced IgE production. An alternative contribution therefore should be considered. CD40L expression on T cells is required for B cells to produce IgE, and the expression of CD40L on T cells can be enhanced by IL-2 receptor signals 3, 27. We therefore assessed whether IL-33 could up-regulate the expression of CD40L and CD25 (IL-2 receptor α chain) on T cells. Because murine CD4^+^ T cells only respond to IL-4 but not IL-13 (because of the lack of IL-13 receptor) 24, we examined IL-33-mediated T-cell function in IL-4^−/−^ mice *ex vivo*. IL-33 increased the surface expression of CD40L and CD25 on CD4^+^ T cells in WT mice but not in IL-4^−/−^ mice (Fig. 4C). Consistent with our finding (Fig. 3C), IL-33 also increased the total number of B cells (but not T cells) in the WT but not in IL-4^−/−^ mice (Fig. 4D). Thus, IL-33 stimulates CD40L and CD25 expression on T cells via IL-4, which may contribute to the IL-33-induced IgE production in naïve mice.

### IL-33 triggers mast cell degranulation in WT but not ST2^−/−^ or IL4Rα^−/−^ mice

Because natural IgE can bind to mast cell FcεRI and impact mast cell functions 5–7, we sought to investigate what effect IL-33 and IL-33-derived IgE may have on mast cell function in naïve mice.

**Figure 5:**
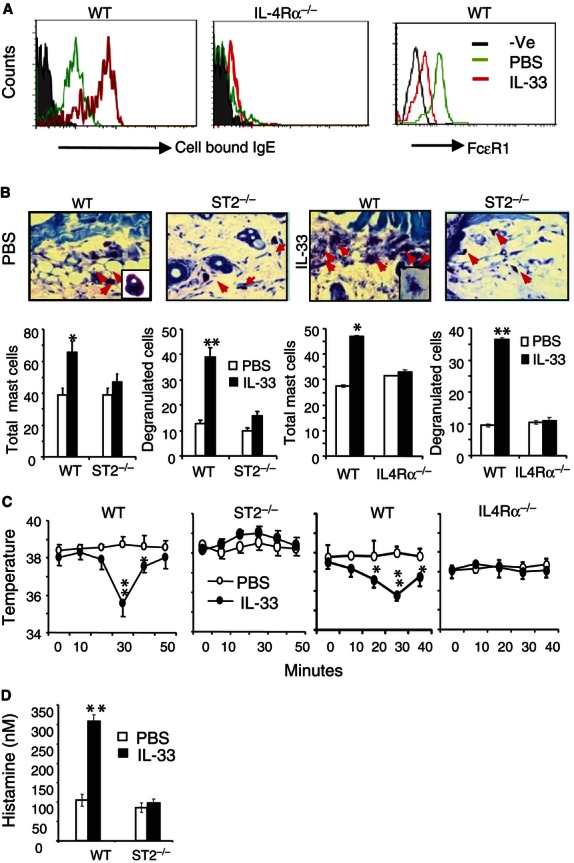
IL-33 triggers anaphylaxis in naïve mice in the absence of allergen. Wild-type (WT), ST2^−/−^ or IL-4Rα^−/−^ mice were injected with IL-33 for 3 consecutive days. (A) The levels of peritoneal c-Kit^+^ mast cell surface IgE and FcεR1 were determined by flow cytometry using specific antibodies. (B) Skin biopsies were stained with toluidine blue to determine the number of degranulated mast cells (indicated by arrows). (C) WT, ST2^−/−^ or IL-4Rα^−/−^ mice were injected i.p. for 3 consecutive days with IL-33 or PBS, and their rectal temperature was determined for up to 50 min postinjection. (D) Mice were killed at the end of 50 min after the third IL-33 injection, and the histamine levels were determined by ELISA. Data are pooled from 3 experiments, *n* = 15 mice per group, *P < 0.05, **P < 0.01 compared to PBS-treated mice.

Naïve WT, ST2^−/−^ or IL4Rα^−/−^ mice were injected with IL-33 or PBS daily for up to 3 days. IL-33 treatment markedly increased the mast cell surface IgE in naïve WT but not in IL4Rα^−/−^ mice as evidenced by enhanced levels of membrane-bound IgE and reduced levels of unoccupied FcεRI on mast cells (Fig. 5A). IL-33 treatment enhanced total and degranulated skin mast cells compared with PBS controls in WT but not ST2^−/−^ or IL4Rα^−/−^ mice (Fig. 5B). More importantly, 30 min after the third IL-33 challenge, WT but not ST2^−/−^ or IL4Rα^−/−^ mice showed clear signs of shock (piloerection, prostration, reduced response to stimuli), with a rapid reduction in body temperature lasting for about 15 min before returning to normal (Fig. 5C), and a substantial increase in serum histamine levels (Fig. 5D). Together, these results demonstrate that IL-33 can initiate mast cell degranulation and systemic anaphylaxis in naïve mice via IL4Rα.

## Discussion

**Figure 6:**
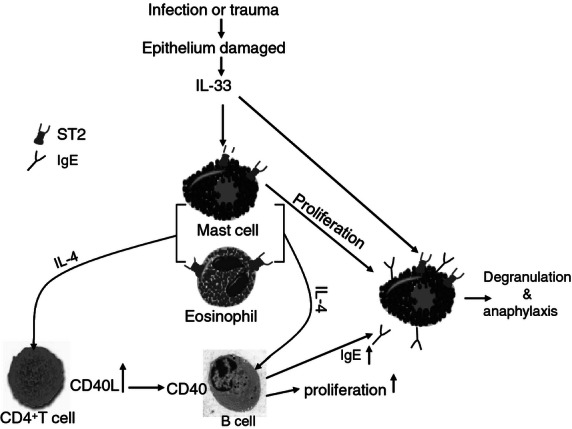
Schematic representation of the mechanism of IL-33-induced IgE synthesis and anaphylaxis in naive mice. IL-33 binding to ST2^+^ innate cells, such as mast cells and eosinophils, leads to mast cell proliferation and increased IL-4 synthesis. IL-4 would then activate B cells to proliferate and to produce IgE. IL-4 produced by the innate cells could also activate T cells to express CD40L that interacts with CD40 on B cells to further enhance IgE production. IgE, together with IL-33 and IL-4, would stimulate mast cells to degranulate, resulting in anaphylaxis.

Data reported in this study reveal a hitherto unrecognized effect and mechanism by which IL-33 induces IgE production and anaphylaxis in the absence of specific allergen (Fig. 6). IL-33 triggers IL-4 secretion by innate cells, which then stimulates B-cell expansion and IgE synthesis. In parallel, IL-4 enhances T-cell expression of CD40L, which interacts with CD40 on B cells to complement IgE production. IL-33 and IgE then synergistically trigger mast cell degranulation and anaphylaxis. Thus, IL-4 is the key intermediate in IL-33-mediated anaphylaxis *in vivo*. Because the mice were allergen naïve and we found that serum samples even from 7 daily injections of IL-33, which showed enhanced levels of total IgE, failed to show any detectable recombinant IL-33-specific IgE (data not shown) by ELISA (Data S1), it is likely, although not proven, that this IgE is natural IgE as described previously 3.

We demonstrated here that eosinophils and mast cells can produce IL-4 upon IL-33 treatment in naïve mice. However, it is likely that other ST2^+^ innate cells such as basophils and NKT cells can similarly produce IL-4 10, 14, 16. By contrast, T cells, which are the key cells expressing type-II cytokines in allergy, are not the major IL-4 producers in this innate immune context. This may be because naïve T cells do not express ST2 and hence are not responsive to IL-33 in the absence of T cell receptor activation 17, 18. In addition, it is known that IL-33 induces mainly IL-5 but not IL-4 in activated T cells and therefore cannot polarize Th2 cells 17.

Using nude mice and T-IL-4Rα-deficient mice, we demonstrated that T cells nevertheless play a significant role in IL-33-derived IgE production in naïve mice. However, this process is unlikely to involve IL-4 production, because nude mice and T-IL-4Rα-deficient mice produced normal or elevated levels of IL-4 and yet had significantly less IgE compared to WT mice. Previous studies have shown that T cells expressing CD40L are required for the induction of natural IgE and that the CD40L expression can be enhanced by IL-2 3, 27. Furthermore, IL-33 can induce IL-2 production in T cells 10. We show here that IL-33 can increase CD40L and CD25 expression on CD4^+^ T cells via IL-4. It is therefore likely that IL-33-derived IL-4 and IL-2, alone or together, stimulate CD4^+^ T cells to express surface CD40L, which subsequently interacts with B cells to promote IgE production.

Anaphylaxis (AS) is an acute, life-threatening allergic reaction in which IgE-mediated mast cell degranulation plays a critical role 28, 29. The aetiology of AS in particular idiopathic AS is not fully understood 28, 30. Our result demonstrated for the first time that IL-33/ST2 signals can trigger anaphylaxis in naïve mice in the absence of specific allergen (Fig. 5). While the detailed molecular mechanism is still unresolved, we found the IL-33-mediated allergen-independent mast cell degranulation and systemic AS described here was dependent on ST2, IL-4Rα and required repeated IL-33 stimulation (Fig. 5C). Thus, the mechanism may comprise a combination of the following two components:

1. It is known that increased mast cell surface IgE expression and mast cell activation are the hallmark of mast cell degranulation and AS. We found that IL-33 can markedly enhance the levels of serum and mast cell surface IgE that is dependent on ST2 and IL-4Rα in antigen-naïve mice. In addition, mast cells also express high levels of ST2, IL-4Rα and IgE FcεR, and it is known that signals via these receptors play critical roles in mast cell activation and function. IL-33 can directly stimulate mast cell proliferation, cytokine production and activation (Fig. 2B, C and ref. 19), and the signals from the interaction between IL-4/IL-13 and IgE with their receptors can activate and prolong the survival of mast cells as well as can exacerbate AS 6, 8, 31.
2. We also found that at least three daily IL-33 treatments were required for IL-33 to trigger mast cell degranulation and AS. Intriguingly, this was also the critical time required for IL-33 to elicit significant production of IgE and IL-4/IL-13 and mast cell proliferation (Figs 1A, 2B,C and 5).

Thus, our results suggest that while IL-33, IL-4 or IgE alone is insufficient to trigger mast cell degranulation without allergen, the accumulated number and activation status of mast cells, strong FcεR signals derived from continued and intensive interaction between IgE and its high-affinity receptor and repetitive IL-33/ST2 and IL-4Rα signals may cumulatively or synergistically reach a signal threshold that leads to mast cell degranulation and AS.

IL-33 is clearly detected in various clinical allergic diseases including asthma and dermatitis 15, 32, 33. Given that many allergic reactions occur without a known allergen, including nonatopic asthma and idiopathic anaphylaxis 30, 34, IL-33 may play a critical role in these diseases. Supporting this hypothesis, we have measured serum IL-33 levels in atopic and nonatopic patients with severe asthma by ELISA. Our results showed that asthma subjects have a wide range of serum IL-33 levels and that there was no significant difference between atopic and nonatopic subjects (Fig. S1). This result is consistent with our finding in the animal model, as well as with a recent report that IL33 polymorphism is correlated with clinical asthma but not necessarily atopy 35.

Because IL-4 and IL-13 also play a pivotal role in antigen-specific IgE production, IL-33 may therefore have a general effect on all IgE-mediated allergic conditions. This concept suggests that IL-33 may represent a novel therapeutic target for a range of allergic diseases.

## Abbreviations

i.p.: intraperitoneal
FcεRI: IgE-Fc epsilon receptor I
AS: anaphylactic shock

## Supporting Information

**Figure S1.** Serum IL-33 levels are not associated with atopy.
